# The optimal timing of frozen-thawed embryo transfer: delayed or not delayed? A systematic review and meta-analysis

**DOI:** 10.3389/fmed.2023.1335139

**Published:** 2024-01-15

**Authors:** Yu-Qi Gao, Jing-Yan Song, Zhen-Gao Sun

**Affiliations:** ^1^The First Clinical College, Shandong University of Traditional Chinese Medicine, Jinan, China; ^2^Reproductive and Genetic Center, The Affiliated Hospital of Shandong University of Traditional Chinese Medicine, Jinan, China

**Keywords:** *in vitro* fertilization, frozen-thawed embryo transfer, immediate, delayed, live birth

## Abstract

**Background:**

The use of frozen embryo transfer (FET) has grown exponentially over the past few years. However, in clinical practice, there are no specific criteria as to whether a delay of at least one menstrual cycle is required for an FET after a failed fresh ET or a freeze-all cycle.

**Objective:**

Through the effects on live birth rate (LBR), clinical pregnancy rate (CPR) and pregnancy loss rate (PLR), to determine whether FET requires a delay of at least one menstrual cycle after fresh ET failure or a freeze-all cycle.

**Methods:**

The search was conducted through PubMed, Web of Science, CNKI, and Wanfang databases for terms related to FET timing as of April 2023. There are no restrictions on the year of publication or follow-up time. Women aged 20 to 46 with any indication for *in vitro* fertilization and embryo transfer (IVF-ET) treatment are eligible for inclusion. Oocyte donation studies are excluded. Except for the case report, study protocol, and abstract, all original studies are included.

**Results:**

In 4,124 search results, 19 studies were included in the review. The meta-analysis includes studies on the adjusted odds ratio (OR) and 95% confidence interval (CI) of reported live birth rate (LBR), clinical pregnancy rate (CPR), and pregnancy loss rate (PLR), 17 studies were retrospective cohort study, and 2 studies were randomized controlled trial, a total of 6,917 immediate FET cycles and 16,105 delayed FET cycles were involved. In this meta-analysis, the combined OR of LBR was [OR = 1.09, 95% CI (0.93–1.28)], the combined OR of CPR was [OR = 1.05, 95% CI (0.92–1.20)], and the combined OR of PLR was (OR = 0.96, 95% CI 0.75–1.22). There was no statistical significance between the two groups.

**Conclusion:**

Overall, delaying FET by at least one menstrual cycle has no advantage in LBR, CPR, or PLR. So, flexible scheduling of FETs is available to both doctors and patients.

**Systematic review registration:**

https://www.crd.york.ac.uk/PROSPERO/, identifier CRD42020161648.

## Introduction

The number of FET cycles in assisted reproductive technology (ART) has been increasing yearly, and it is estimated that in 2014, FET accounted for approximately 40% of the approximately 2 million ART treatment cycles per year worldwide (1). In fact, with the advancement and improvement of freezing, thawing, and resuscitation techniques, frozen embryos are almost indistinguishable from fresh embryos in terms of quality and implantation potential (2, 3). In cases where fresh embryo transfers fail or in cases where fresh embryos fail to transfer for various reasons, patients choose FET.

After determining to adopt FET, how far apart does FET need to be performed for optimal clinical outcomes? The use of controlled ovarian stimulation (COS) before IVF is mostly aimed at obtaining more embryos and, consequently, increasing the success rate of the procedure. Nevertheless, concerns have been raised about the adverse effects of supraphysiological hormones used in COS, including embryo-endometrial asymmetry (4) and alteration of the endometrium’s immune system (5), which may adversely affect the pregnancy outcome of subsequent embryo transfers. There are also multiple luteal or luteal cysts after oocyte retrieval and functional cysts may lead to ovulation disorders and increase the cancellation rate of the FET cycle. If immediate FET fails, the pressure and economic burden on patients will be increased. Therefore, in current clinical practice, most ET procedures are delayed, a practice that aims to minimize the possible residual negative effects of COS on the recovery to normal ovulatory cycles and endometrial receptivity.

However, it has not yet been determined whether delaying FET leads to a better outcome. As a social issue, infertility is a major problem that cannot be ignored, and it also causes heavy psychological stress to patients. In addition, negative emotions such as excessive anxiety and depression can have a negative impact on pregnancy outcomes (6, 7). For infertile couples, delayed ET is a challenge and should be further explored to minimize interruptions in treatment. Therefore, the purpose of this study is to determine whether FET should be delayed for at least one menstrual cycle following a failed fresh ET or following a freeze-all cycle.

## Materials and methods

### Inclusion criteria and exclusion criteria

#### Inclusion criteria

1. Study design: randomized controlled trial or cohort study.
2. Participants: women who underwent their first FET following failed fresh ET or freeze-all cycle.
3. Outcome measures: CPR, LBR, and PLR are the primary outcomes of interest.

#### Exclusion criteria

1. Those who have undergone preimplantation genetic diagnosis and screening (PGD/PGS).
2. Patients who have not undergone an ovarian stimulation cycle.
3. Repeated publication, incomplete data, unable to obtain the full text.
4. Studies on oocyte donation.

### Search strategy

We searched PubMed, Web of Science, CNKI, Wanfang, and other databases for medical subject titles as of April 2023, as well as text words related to FET timing. In addition, the references of the included literature were searched to supplement the acquisition of relevant information. The search method is a combination of free words and subject words. The search terms included “freeze all,” “fresh embryo transfer,” “infertility,” “frozen embryo transfer” or “frozen-thawed embryo transfer” or “cryopreserved embryo transfer,” “immediate” or “delayed” or “postpone,” “timing” or “time” or “time interval,” “oocyte retrieval” or “ovum pick-up,” “ovarian stimulation,” “IVF” or “Fertilization *in Vitro*” or “OPU” etc.

### Data extraction

For data extraction, the two researchers independently read the literature based on the unified inclusion and exclusion criteria. In case of disagreement, the third researcher will participate in the discussion and decide. Information extracted included first author’s name, year of publication, country of origin, study design, population characteristics, definition of immediate/delayed FET, ovarian stimulation protocol, trigger agent, endometrial preparation protocol, embryonic development stage, and outcome parameters.

### Risk of bias evaluation

The Newcastle–Ottawa scale (NOS) was used to evaluate the methodological quality of the eligible studies. The scale assigns a maximum of 9 points to each study based on three broad dimensions: subject selection and exposure assessment (4 points), comparability of study groups (2 points), and adequacy of outcome ascertainment and follow-up (3 points). studies with a score of 7–9 are of high quality and low risk of bias. The investigators scored each study independently, and discrepancies were resolved by consensus with the third investigator. The Cochrane Handbook was used to evaluate the methodological quality of the eligible studies. The evaluation content consists of 7 items. Each entry was rated as “low risk,” “unknown,” and “high risk.”

### Statistical methods

Using RevMan 5.4 statistical software. Relative risk (RR) and 95% CI were selected as the statistical variables of binary classification. Mean difference (MD) and its 95% CI were selected as statistical variables for continuity variables. The statistical heterogeneity of the included studies was analyzed and judged by *p*-value and *I*^2^. When *p* > 0.1 and *I*^2^ ≤ 50%, the heterogeneity among the studies was small, and the fixed-effect model was used for meta-analysis. When *p* ≤ 0.1 or *I*^2^ > 50%, it indicates that there is a large heterogeneity among studies, and a random effects model is used. When the heterogeneity was large, sensitivity analysis was carried out by eliminating each study one by one to check whether the results were stable, and descriptive analysis was carried out to explore the possible sources of heterogeneity. Test level *α* = 0.05.

## Result

**Figure 1:**
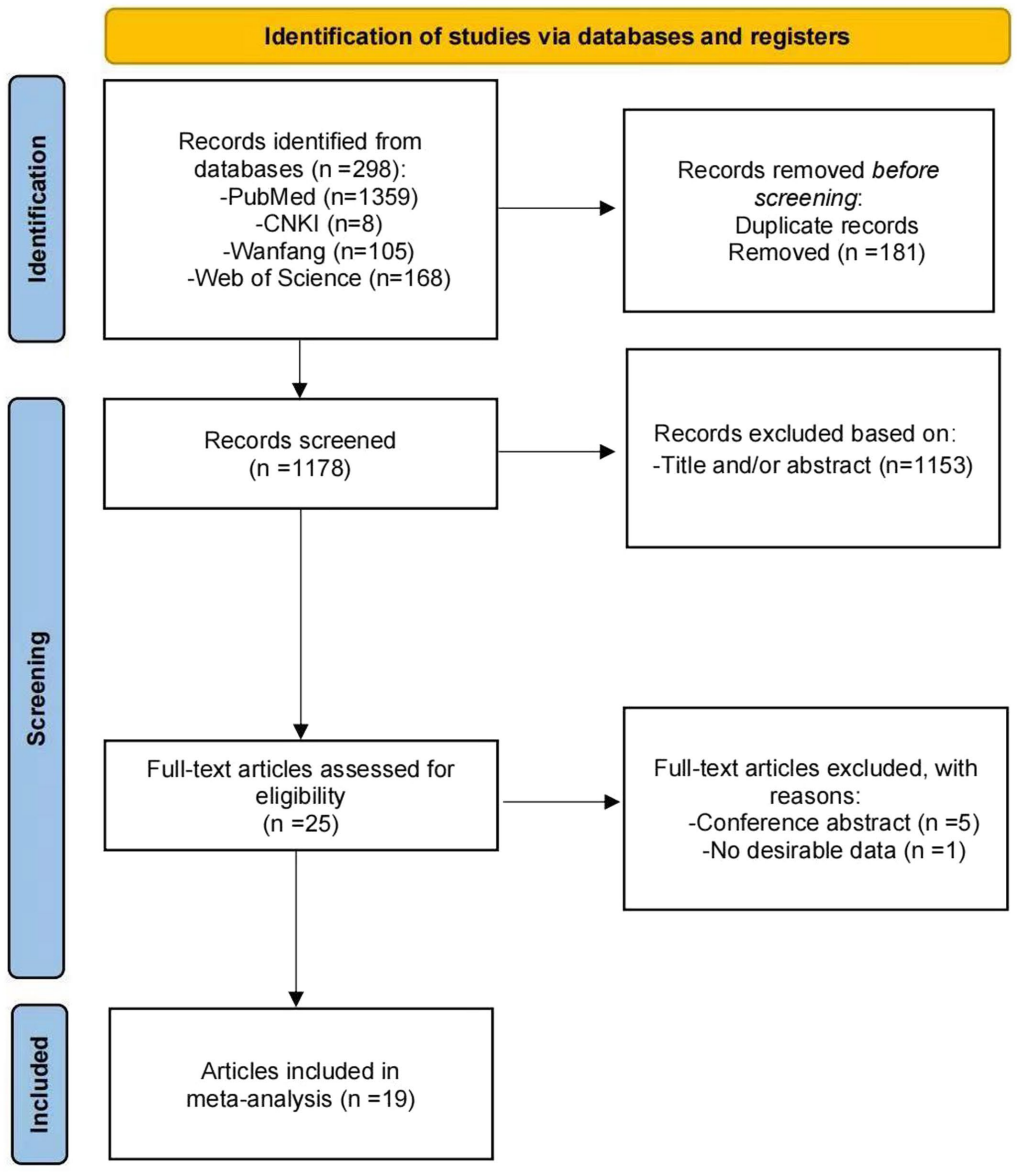
PRISMA flow diagram of search and selection strategy.

A total of 19 studies were included in this systematic review (8–26). All 17 studies were retrospective cohort studies and 2 were randomized controlled trials. The studies included a total of 23,111 cycles, of which 6,842 immediate FETs and 16,269 delayed FETs were involved. The flow chart of literature retrieval is shown in Figure 1, and the general information and quality evaluation results of the included literature are shown in Tables 1-3.

**Table 1:** The basic information of included studies.

Study	Country	Publication date	Study design	Definition of immediate/delayed FET	Population	Embryonic development stage	Trigger agent	Ovarian stimulation protocol	Endometrial preparation	Outcome
Lattes 2016	Spain	2016	Retrospective cohort study	<1 cycle/≥ 2 cycles from oocyte retrieval to the start of FET	Freeze-all	Cleavage stage	GnRHa /dual trigger	GnRH-ant protocol/long GnRH agonist protocol	HRT	LBR, CPR, PLR
Chen 2019	China	2019	Retrospective cohort study	<1 cycle/≥ 2 cycles from oocyte retrieval to the start of FET	Freeze-all	/	hCG	Super long protocol/long GnRHa protocol/short GnRHa protocol/GnRH-ant protocol	HRT	LBR
He 2020	China	2020	Retrospective cohort study	<1 cycle/≥ 2 cycles from oocyte retrieval to the start of FET	Freeze-all	Cleavage and blastocyst stage	hCG	GnRH-ant protocol/GnRHa pituitary down-regulation protocol	HRT/NC	LBR, CPR
Higgins 2017	Australia	2017	Retrospective cohort study	25–35/50–70 days cycles from oocyte retrieval to the start of FET	Freeze-all	Blastocyst stage	hCG	GnRH-ant protocol/GnRHa pituitary down-regulation protocol/GnRHa protocol	HRT/NC	CPR, LBR, PLR
Horowitz 2019	Israel	2019	Retrospective cohort study	<22/≥ 22 days from failed IVF-ET cycle to FET	Failed fresh ET	Cleavage and blastocyst stage	hCG	GnRH-ant protocol/GnRHa protocol	NC	CPR, LBR
Hu 2020	China	2020	Retrospective cohort study	≤40/> 40 days from oocyte retrieval to the start of FET	Freeze-all	Blastocyst stage	hCG	GnRH-ant protocol/GnRHa protocol	HRT	CPR, LBR, PLR
Huang 2019	China	2019	Retrospective cohort study	<1 cycle/≥ 2 cycles from oocyte retrieval to the start of FET	Freeze-all	Cleavage and blastocyst stage	hCG/GnRHa agonist/dual trigger	Progestin primed ovarian stimulation protocol, short GnRHa protocol	HRT + NC	CPR, LBR
Kaye 2017	United States	2017	Retrospective cohort study	<1 cycle/≥ 2 cycles from oocyte retrieval to the start of FET	Freeze-all	Blastocyst stage	hCG/GnRHa agonist/Dual trigger	GnRH-ant protocol/GnRHa protocol	HRT + NC	CPR, LBR
Yildiz 2021	Turkey	2021	Retrospective cohort study	≤30/> 30 days from oocyte retrieval to the start of FET	Freeze-all	Blastocyst stage	hCG/GnRHa agonist/Dual trigger	Progestin primed ovarian stimulation protocol, short GnRHa protocol	HRT	LBR
Li 2021	China	2021	Randomised controlled trial	<1 cycle/≥ 2 cycles from oocyte retrieval to the start of FET	Failed fresh ET and freeze-all cycle	Cleavage and blastocyst stage	hCG/GnRHa agonist/dual trigger	Long GnRHa protocol/GnRH-ant protocol	HRT	LBR, CPR
Liang 2017	China	2017	Retrospective cohort study	≤45/> 45 days from oocyte retrieval to the start of FET	Freeze-all	Cleavage and blastocyst stage	hCG	GnRH-ant protocol/GnRHa protocol	HRT/NC	CPR, LBR
Peng 2019	China	2019	Retrospective cohort study	<1 cycle/≥2 cycles from oocyte retrieval to the start/failed IVF-ET cycle of FET	Failed fresh ET and freeze-all cycle	Cleavage and blastocyst stage	hCG	GnRHa pituitary down-regulation protocol	HRT/NC	CPR
Samuel Santos-Ribeiro 2016 (1)	Brussel	2016	Retrospective cohort study	≤22/> 22 days from failed IVF-ET cycle to FET	Failed fresh ET	Cleavage and blastocyst stage	HCG	GnRH-ant protocol	HRT/NC	CPR, LBR
Samuel Santos-Ribeiro 2016 (2)	Brussel	2016	Retrospective cohort study	<1 cycle/≥ 2 cycles from oocyte retrieval to the start of FET	Freeze-all	Cleavage and blastocyst stage	hCG	GnRH-ant protocol	HRT	CPR
Song 2019	China	2019	Retrospective cohort study	<1 cycle/≥ 2 cycles from oocyte retrieval to the start of FET	Freeze-all	Cleavage stage	hCG/GnRHa agonist/dual trigger	GnRH-ant protocol/GnRHa protocol/mini-stimulation protocol/GnRHa pituitary down-regulation protocol	HRT/NC	LBR
Song 2021	China	2021	Randomised controlled trial	<1 cycle/≥ 2 cycles from failed IVF-ET cycle to FET	Failed fresh ET	Cleavage stage	hCG	GnRH-ant protocol	HRT	CPR, PLR, LBR
Tian 2021	China	2020	Retrospective cohort study	<90/≥ 90 days from failed IVF-ET cycle to FET	Failed fresh ET	Cleavage and blastocyst stage	hCG	GnRH-ant protocol/GnRHa protocol	HRT/NC	CPR
Volodarsky-Perel 2016	Israel	2020	Retrospective cohort study	<50/≥ 50< 120 days from failed IVF-ET cycle to FET	Failed fresh ET	Cleavage and blastocyst stage	hCG	Long GnRH-agonist protocol	HRT	CPR, LBR
Xu 2021	China	2020	Retrospective cohort study	≤1 cycle/> 2 cycles/> 3 cycles from oocyte retrieval to the start of FET	Failed fresh ET	Cleavage stage	hCG	CC + hMG ovulation induction protocol	HRT	CPR, LBR

FET, frozen embryo transfer; ET, embryo transfer; LBR, live birth rate; CPR, clinical pregnancy rate; PLR, pregnancy loss rate; HRT, hormone replacement therapy; NC, natural cycle; GnRH, gonadotropin releasing hormone; hCG, human chorionic gonadotrophin; CC, clomiphene citrate; hMG, human menopausal gonadotropin.

**Table 2:** Newcastle–Ottawa scale for assessing the quality of studies in meta-analysis.

Study	Selection				Comparability	Outcomes			Quality Score
	Representativeness of the exposed cohort	Selection of the non-exposed cohort	Ascertainment of exposure	Demonstration that outcome of interest was not present at start of study	Comparability of cohorts on the basis of the design or analysis	Assessment of outcome	Was follow-up long enough for outcomes to occur	Adequacy of follow up of cohorts	
Lattes 2016	1	1	0	0	2	0	1	1	6
Chen 2019	1	1	0	0	2	0	1	1	6
He 2020	1	1	1	0	2	1	1	1	8
Higgins 2017	1	0	1	0	2	1	1	1	7
Horowitz 2019	1	1	0	0	2	0	1	1	6
Hu 2020	1	1	0	0	2	0	1	1	6
Huang 2019	1	1	0	0	2	0	1	1	6
Kaye 2017	1	1	1	0	2	0	1	1	7
Yildiz 2021	1	1	1	0	2	1	1	1	8
Li 2021	1	1	0	1	2	1	1	1	8
Liang 2017	1	1	0	0	2	0	1	1	6
Peng 2019	1	1	0	0	2	0	1	1	6
Samuel Santos-Ribeiro2016 (1)	1	1	0	0	2	0	1	1	6
Samuel Santos-Ribeiro2016 (2)	1	1	0	0	2	0	1	1	6
Song 2019	1	1	1	0	2	1	1	1	8
Song 2021	1	1	0	2	2	1	1	1	9
Tian 2021	1	1	1	0	2	1	1	1	8
Volodarsky-Perel 2016	1	1	1	0	2	1	1	1	8
Xu 2021	1	1	1	0	2	1	1	1	8

### Meta-analysis of CPR

**Figure 2:**
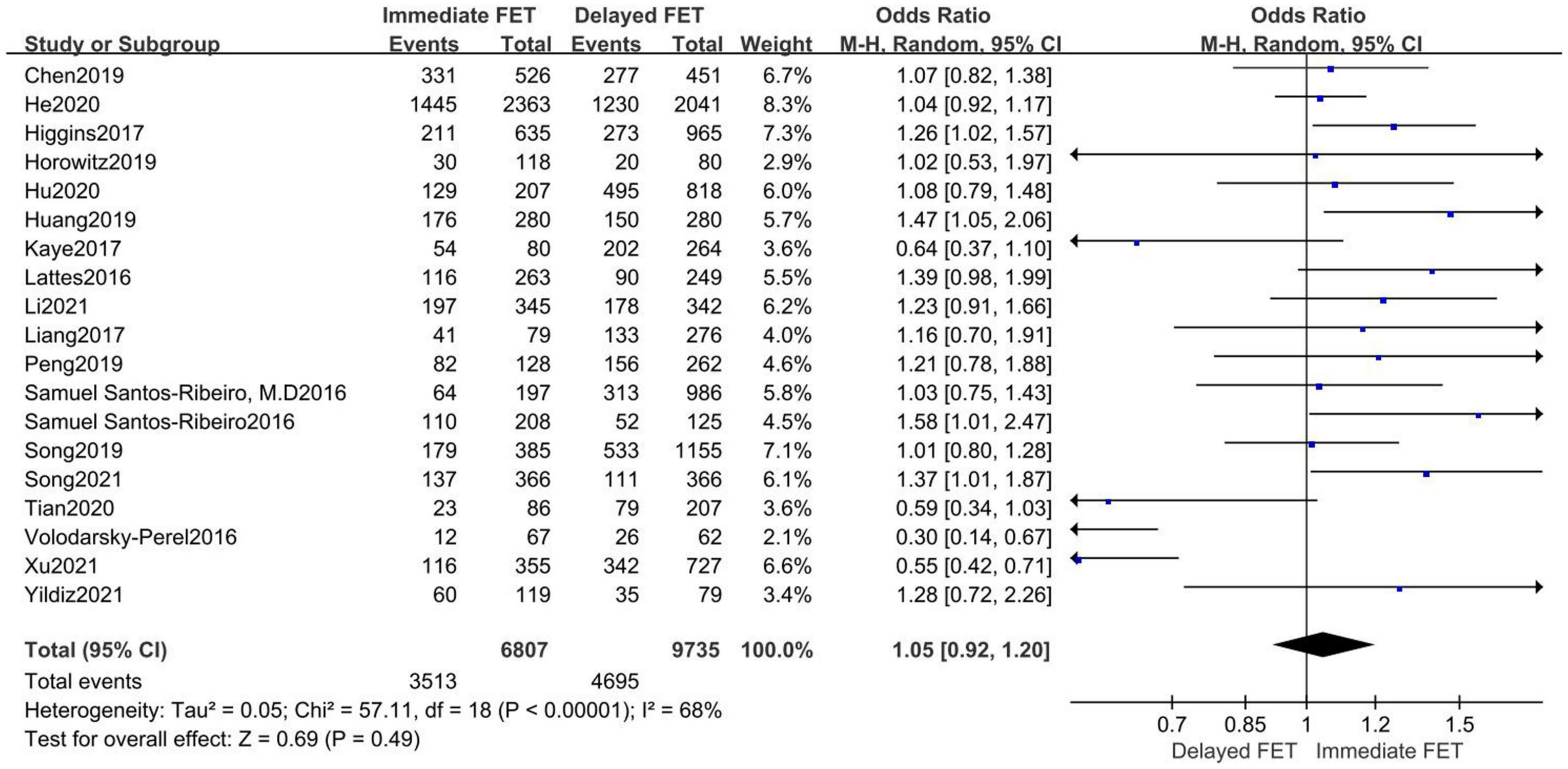
Forest plots of the association between immediate FET and delayed FET and clinical pregnancy rates.

**Figure 3:**
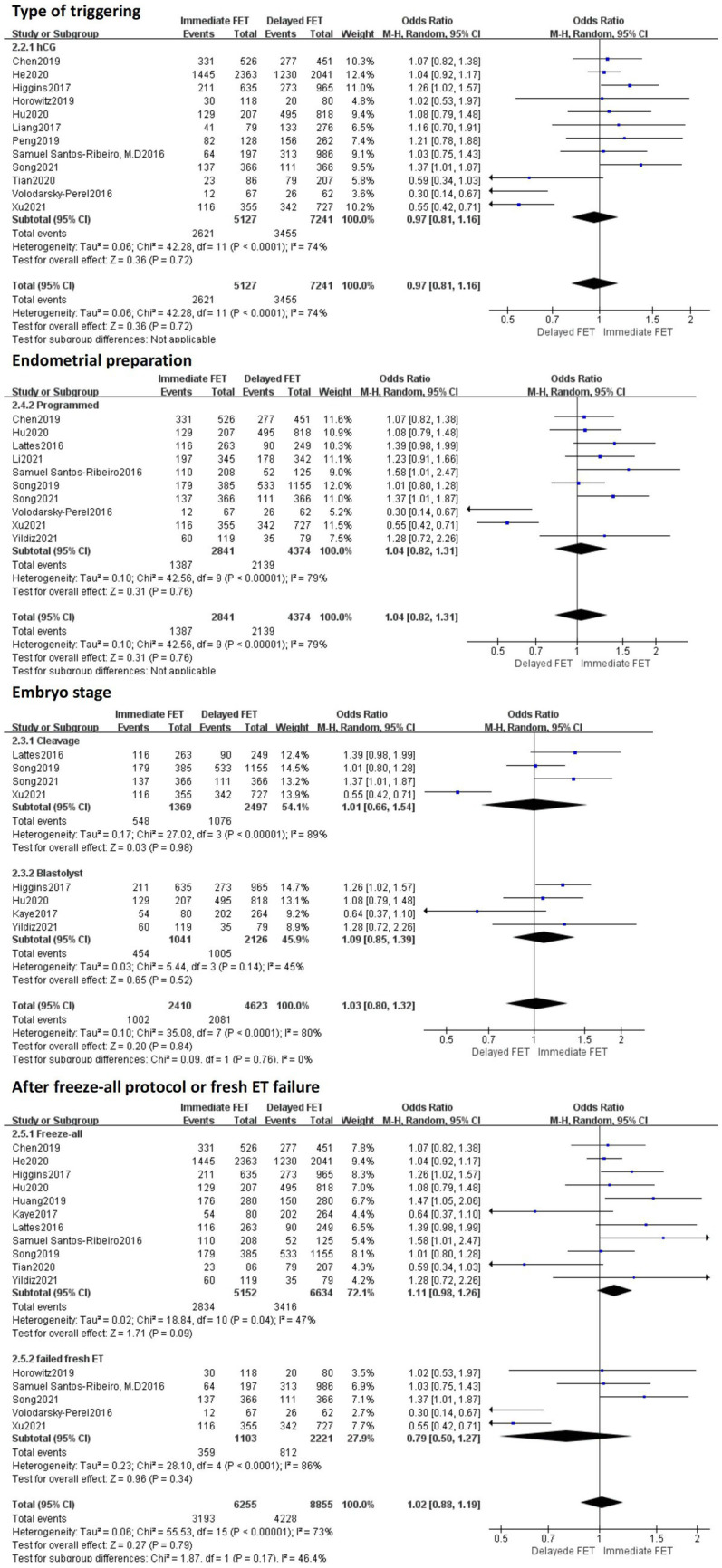
Subgroup analysis of clinical pregnancy rate.

A total of 19 literatures with CPRs supported by original data were included. The combined results of these studies showed that there was no statistical significance in CPR between the immediate FET group and the delayed FET group [OR = 1.05, 95% CI (0.92–1.20), *p* > 0.05] (Figure 2). We believe that immediate FET is not superior to delayed FET in CPR. In addition, the included studies are highly heterogeneous. To determine the source of heterogeneity, we conducted multi-group subgroup analysis. The subgroup analysis of type of triggering (OR 0.97, 95% CI 0.81–1.15), embryo stage at transfer (OR 1.03, 95% CI 0.80–1.32), endometrial preparation (OR 1.04, 95% CI 0.82–1.31), and FET cycle following a freeze-all cycle or fresh ET failure (OR 1.02, 95% CI 0.88–1.19), did not reveal any statistical significance in CPR between the two groups (Figure 3).

### Meta-analysis of LBR

**Figure 4:**
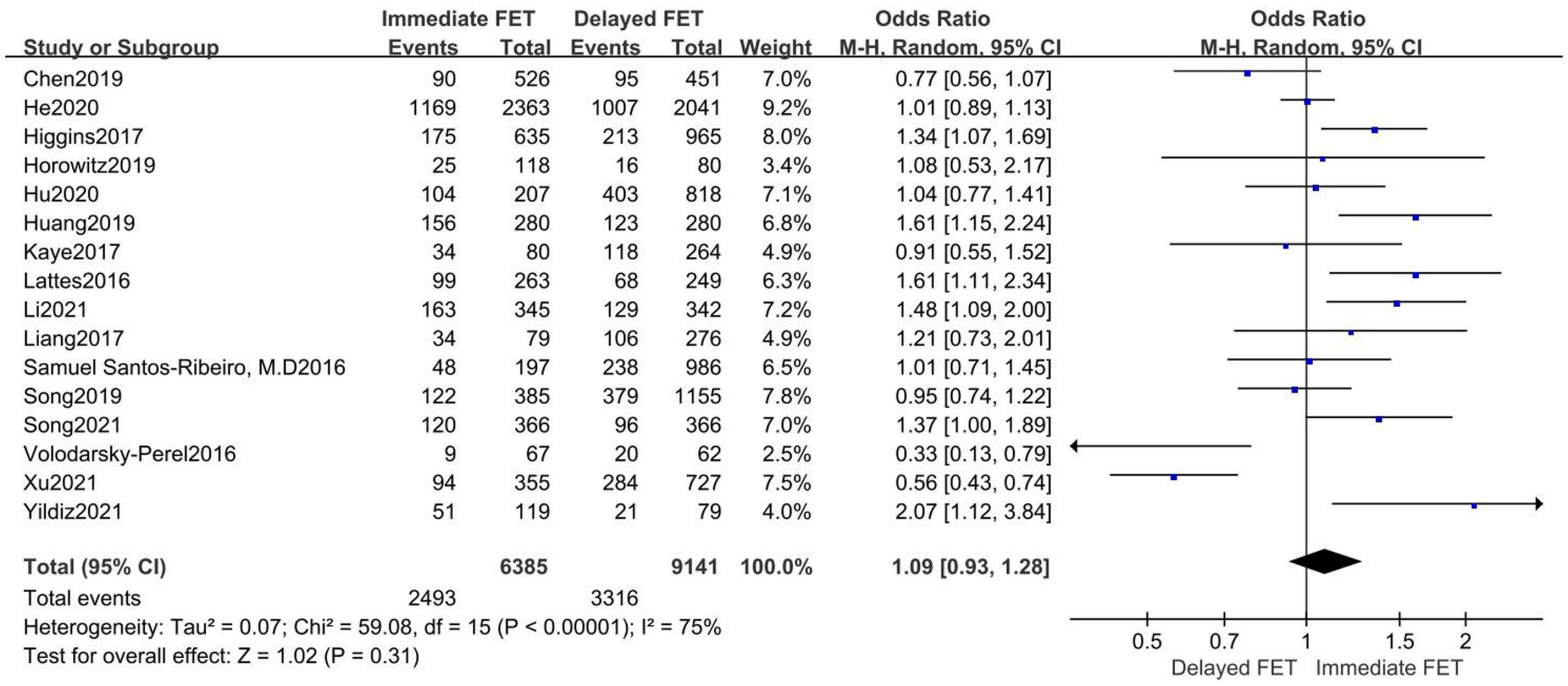
Forest plots of the association between immediate FET and delayed FET and live birth rate.

**Figure 5:**
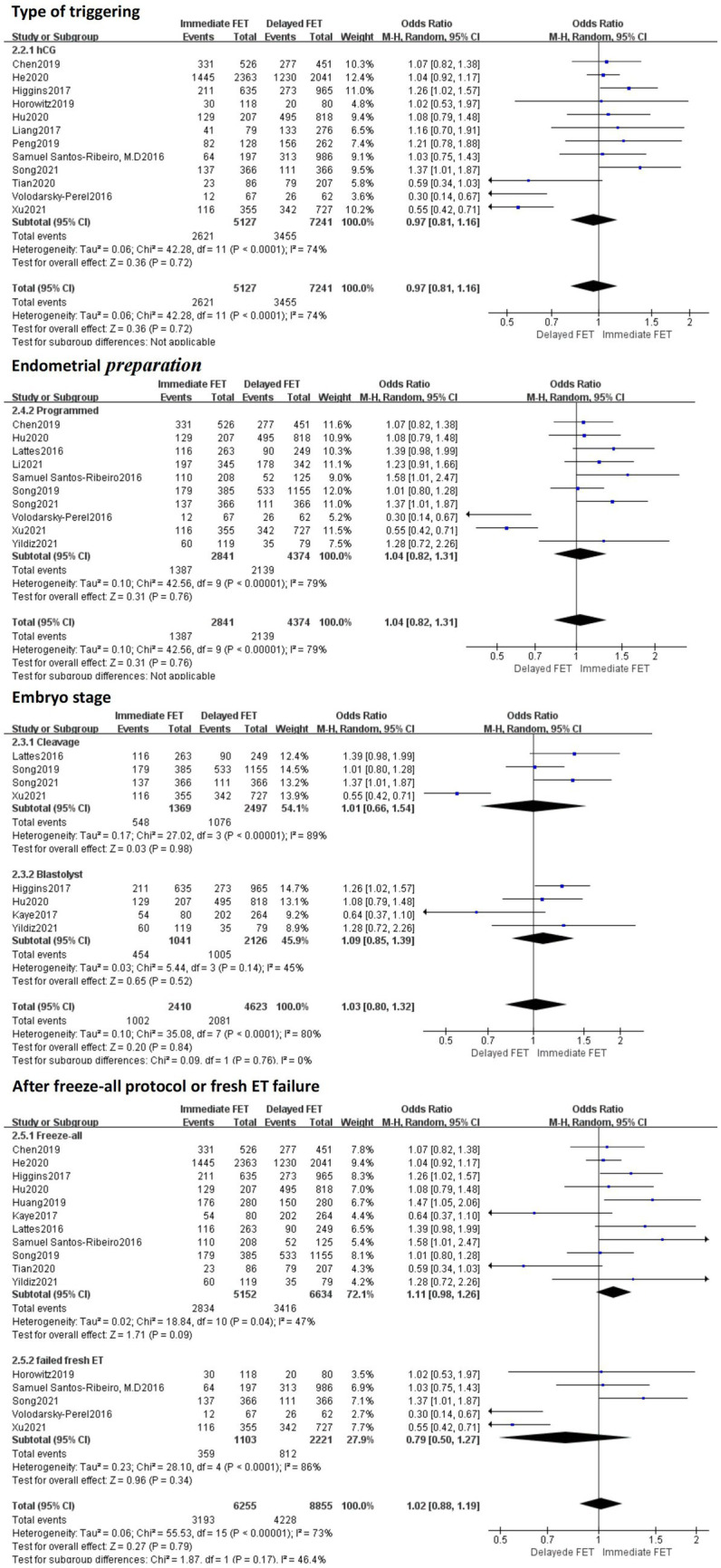
Subgroup analysis of live birth rate.

A total of 16 publications with original data were included. According to Figure 4, there was no statistically significant difference between the immediate and delayed FET groups on LBR [OR = 1.09, 95% CI (0.93–1.28), *p* = 0.31], suggesting that the immediate FET was not superior to the delayed FET in LBR. Considering the high heterogeneity, multi-group subgroup analysis was performed, and the combined result remained unchanged when subgroup analysis was performed for FET cycles following fresh ET failure and for FET cycles following freeze-all (OR 1.05, 95% CI 0.99–1.25). Similarly, subgroup analyses of type of trigger (RR 0.96, 95% CI 0.79–1.17), endometrial preparation (RR 0.97, 95% CI 0.73–1.29), and embryo stage (RR 1.12, 95% CI 0.86–1.46) did not reveal any differences (Figure 5).

### Meta-analysis of PLR

**Figure 6:**
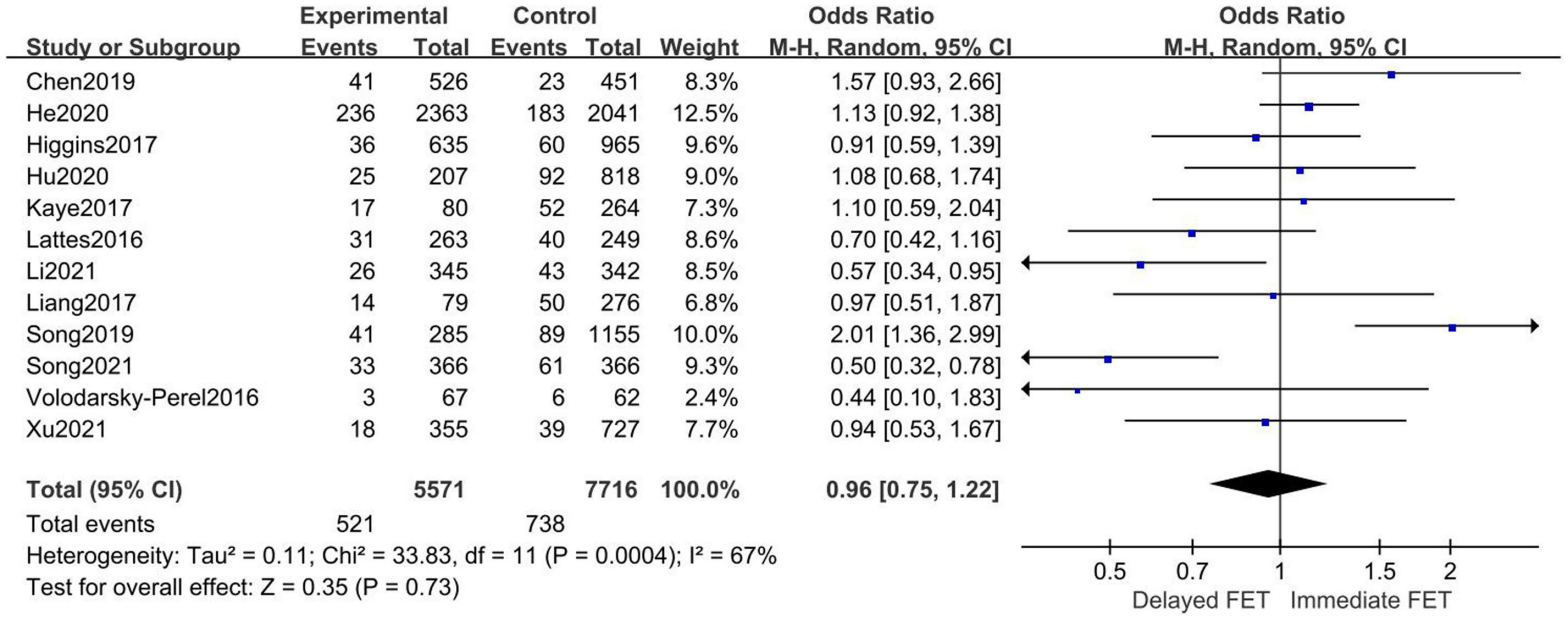
Forest plots of the association between immediate FET and delayed FET and pregnancy loss rate.

**Figure 7:**
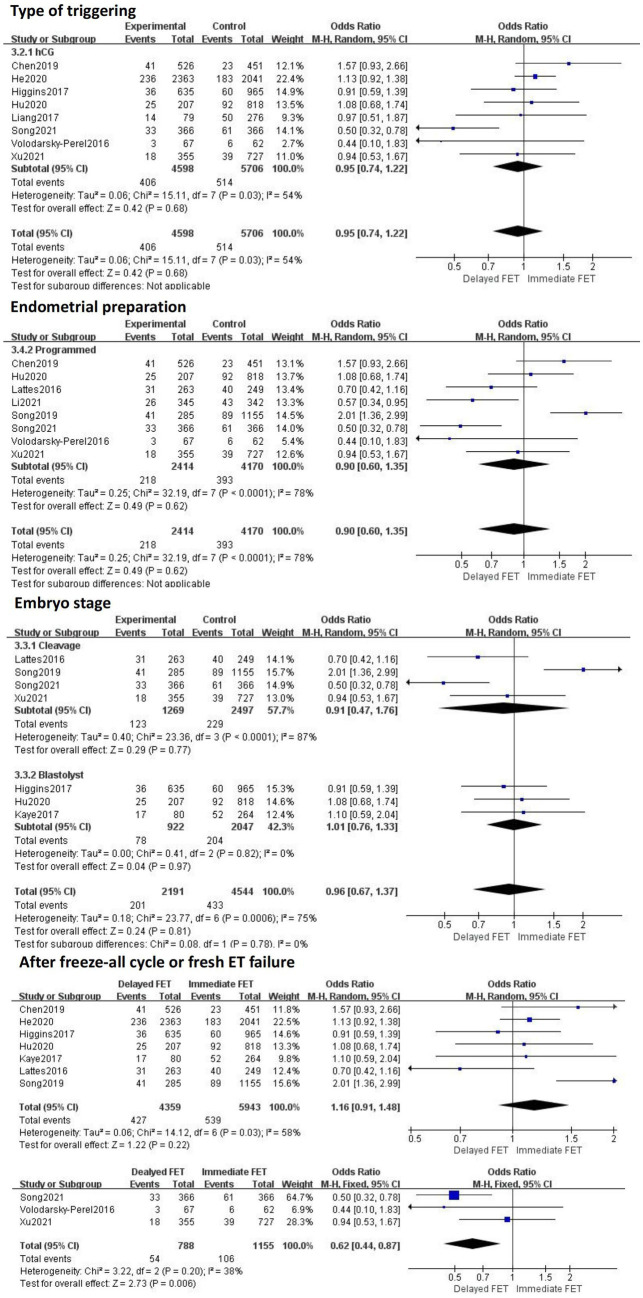
Subgroup analysis of pregnancy loss rate.

A total of 12 literatures were included, as shown in the forest diagram in Figure 6. The results of meta-analysis showed that there was no statistical significance (OR = 0.96, 95% CI 0.75–1.22) between immediate FET and delayed FET groups on PLR. To identify the source of heterogeneity, a multi-group subgroup analysis was performed. Type of triggering (OR 0.95, 95% CI 0.74–1.22), endometrial preparation (OR 0.90, 95% CI 0.60–1.35), and embryo stage (RR 0.96, 95% CI 0.67–1.33) were evaluated (Figure 7). However, in the subgroup analysis, after fresh ET failure, delayed FET had a higher rate of pregnancy loss than immediate FET (OR 0.62, 95% CI 0.44–0.87, see Figure 7).

## Discussion

In this systematic review and meta-analysis, the effects of FET timing on LBR, CPR, and PLR were summarized. In general, the timing of FET, that is, whether it is performed immediately after fresh ET failure or delayed after freeze-all cycles, LBR, CPR, and PLR was not superior to immediate FET. However, in the FET cycle after fresh ET failure, the PLR with immediate FET is lower than that with delayed FET.

Out of 19 studies, our conclusions are consistent with those of 7 studies (9, 14, 15, 17, 19, 24, 25), regardless of which COS protocol is adopted. While FET is not necessary to delay a menstrual cycle after a freeze-all cycle, Yildiz et al. (24) and Hu et al. (12) both suggest delayed FET may result in a higher birth weight, preeclampsia, and macroia, which may result from the loss of corpus luteum during an artificial cycle and an extended period of isolation and freezing of embryos. On the other hand, the results of He’s et al. (9) study showed that there were no significant differences between immediate and delayed FET cycles in terms of preterm birth, gestational age, birth weight, congenital malformations and sex ratio, and that immediate FET did not improve neonatal risk, which needs more research to be confirmed.

Huang et al. (13) and Higgins et al. (10) have different conclusions with us. In their study, they found that immediate FET has a higher LBR than delayed FET. Most of the patients included in Huang’s study underwent COS with exogenous gonadotrophins by using progestin-primed ovarian stimulation (PPOS) or gonadotropin-releasing hormone agonist (GnRH-a) short protocol, and in the author’s opinion, many luteal products after COS can restore the endometrial blood vessels and improve pregnancy outcomes (13). Nevertheless, Kaye et al. (14) suggests delaying one cycle, as immediate FET cycles can indicate a dysfunctional menstrual cycle.

The optimal timing of FET after a failed fresh ET cycle is a common problem, and after subgroup studies, we found that the PLR of immediate FET after fresh ET failure was lower than that of delayed FET. A large number of follicles develop in COS, and the influence of ovarian superphysiological doses of hormones on endometrial receptivity, resulting in embryo-endometrial dissynchrony (27) may make clinicians more inclined to delay FET after fresh ET failure. However, the study by Horowitz et al. (11), Santos-Ribeiro et al. (18), Song et al. (20), Tian et al. (21), and Peng et al. (26) showed that pregnancy outcomes after fresh ET were better than those after delayed FET, whether in the modified natural cycle or hormone replacement cycle. In Song’s et al. (20) study, the frequency of moderate-to-severe depression and high stress level before FET was significantly higher in the delayed FET group than in the immediate FET group, and high stress level and high stress level had adverse effects on continued pregnancy and live birth rate (28).

In contrast, research by Volodarsky-Perel et al. (22) and Xu et al. (23) found a positive effect of delaying FETs. A long GnRH-a regimen was used by Volodarsky-Perel et al. (22), and the effects of GnRH-a on the endometrium in the ovarian hyperstimulation cycle were found to persist into adjacent menstrual cycles. There are studies showing that, after the full dose of GnRH-a is injected, the effect on the menstrual cycle can last for 11–13 weeks (29). Nevertheless, some studies have evaluated the clinical efficacy of long-acting GnRH agonists in general populations, and have identified a variety of proteins that facilitate embryo implantation in the endometrium, suggesting that long-acting agonists may enhance endometrial receptivity (30). In addition, another study showed that increased levels of GnRH-a directly modulate the expression of enzymes and cytokines and increase the expression of endometrial tolerance markers such as integrin b3 and leukaemia inhibitory factor, improving endometrial tolerance and clinical outcome in patients with intermediate and very thin endometrium (31). Xu’s et al. (23) study used clomiphene citrate (CC) + human menopausal gonadotrophin (HMG) protocol for COS. In clinical practice, CC is widely used as a first-line ovulation-promoting drug. However, due to its anti-estrogen effect, CC occupies endometrial estrogen receptors, inhibits endometrial proliferation, promotes endometrial cell apoptosis, and affects endometrial receptivity through various ways. For example, the study compared the expression of key molecules in the Wnt/β-catenin signaling pathway during the CC expulsion cycle, and CC significantly down-regulated Wnt signaling, which led to thinning of the endometrium (32). Furthermore, due to the prolonged use time of CC during the ovulation induction process, it may take longer for metabolism clearance to be completed (33). Furthermore, this study indicates that embryo implantation rates, CPRs and LBRs during the first menstrual cycle after oocyte retrieval are significantly less than those in other groups (23).

In the selected studies, ovarian hyperstimulation syndrome (OHSS) is a common and potentially risky iatrogenic complication. Especially for women with high ovarian response, the risk of acquiring OHSS is higher, and FET after embryo freezing is the most meaningful strategy for these women (34). A study of 2,060 cases found that delaying the FET cycle did not improve live birth rates in patients who cancelled ET because of high risk of OHSS (35). Patients who opt for a freeze-all policy due to OHSS may have relatively good ovarian reserve function, which may optimize the results of an immediate FET. In addition, differences in embryo quality may be a confounding factor in the comparison of clinical outcomes between the two groups, as embryos with the highest implantation potential are usually transferred first according to morphodynamic criteria, so embryos transferred mid-cycle in the delayed FET group may be of poorer quality than those in the immediate FET group.

**Table 3:** Cochrane for assessing the quality of studies in meta-analysis.

Study (randomized controlled trial)	Selection bias		Performance bias	Detection bias	Attrition bias	Reporting bias	Other bias
	Random sequence generation	Allocation concealment	Blinding of participants and personnel	Blinding of outcome assessment	Incomplete outcome data	Selective reporting	Other bias
Li 2021	Low risk of bias	Low risk of bias	High risk of bias	High risk of bias	Low risk of bias	Low risk of bias	Low risk of bias
Song 2021	Low risk of bias	Low risk of bias	High risk of bias	Low risk of bias	Low risk of bias	Low risk of bias	Low risk of bias

Additionally, differences in endometrial preparation protocols between included studies, such as programmed cycle (PC) and natural cycle (NC), may have increased the risk of selection bias. To eliminate potential bias based on the type of endometrial preparation protocol for FETs, we performed a subgroup analysis of PC-FETs, but because most studies in this review were a combination of PC-FETs and NC-FETs, or PC-FETs alone, a subgroup analysis of NC-FETs was not possible. Subgroup analyses of endometrial preparation protocols revealed no significant differences between immediate and delayed PC-FET groups in LBR, CPR, and PRL. PC-FET is a better option for patients with irregular periods, amenorrhoea or poor response to ovulation induction, prolonged persistent anovulation, and recalcitrant polycystic ovary syndrome (PCOS), and PC-FET requires luteal support in the later stage and has strong operability, and patients do not need to be hospitalized for multiple monitoring. NC-FET is a safer and more natural endometrial preparation protocol, in which the timing of embryo transfer is determined by the increased production of luteinizing hormone (LH) or human chorionic gonadotropin (hCG), which induces ovulation. However, women with NC for endometrial preparation must monitor ovulation frequently, and there is a high probability of cycle cancellation, which increases the mental stress and financial costs of the patient. Despite this, studies indicate that NC-FET suffers less complications than PC protocol due to the lack of luteum (36). PC-FET significantly increases the risk of pregnancy-induced hypertension and placental implantation compared to NC-FET. In 2020, Singh et al. (37), summarized recent research on the impact of luteum on FET obstetric outcomes, highlighting the risk for preeclampsia, postpartum hemorrhage, macroia, and overdue labor associated with PC-FET without luteum production, and stating that the luteum plays a crucial role in preventing obstetric complications. In addition to luteal deficiency, Zong’s et al. (38) study found that elevated estrogen levels not only significantly suppressed vascular invasion, but also impaired trophoblast invasion and may be associated with poor maternal and neonatal outcomes. As of now, however, there is no strong evidence supporting which endometrial preparation regimen is more advantageous for women with regular menstrual cycles.

Following fresh ET failure or freeze-all cycles, it may be cumbersome and outdated to delay FET for at least one menstrual cycle in order to minimize the potential negative effects of ovarian stimulation and multiple luteum on the restoration of normal ovulation cycles and the receptive endometrium. Nevertheless, the selected literature does not provide a specific explanation for canceling fresh ET, nor does it provide any explanation for selecting immediate or delayed FET criteria, therefore, in clinical practice, it is imperative that a strict set of delayed FET criteria be established based upon the adverse conditions for immediate FET.

After the development of ART, several studies have demonstrated that the timing of FET following the cancellation of fresh ET does not have a significant impact on pregnancy outcomes. With the advancement in freeze-thaw and resuscitation technology, embryos can be preserved to the maximum extent possible and the quality of freezing and thawing can be improved. In this way, the timing of FET after fresh ET failure or the freeze-all policy has little impact on pregnancy outcomes.

In the present study, it appears that delayed FET may be unnecessary, but caution should be exercised in its interpretation. Important limitations of this review are the retrospective design, including the heterogeneity of the studies. In addition, in some studies, the existence of selection bias is obvious. No article in this systematic review specifically explained the reasons for choosing freeze-all policy instead of fresh ET, the reasons for choosing immediate FET or delayed FET, and the length of time for delayed FET. Therefore, the risk of selection bias is obvious, and the quality of studies is uneven. The results measured in this study included clinical pregnancy, live birth, and preclinical pregnancy loss. Other outcomes, such as preterm birth, birth weight, and fetal development, are not considered, which may also be affected by ovarian stimulation, and therefore by FET timing, and should therefore be considered when applying these results to clinical practice.

## Conclusion

Overall, FET immediately or subsequently after fresh ET failure or freeze-all policy had no adverse effects on pregnancy outcomes. Due to the limited number of retrospective cohort studies evaluated, selection bias was evident, and the overall quality of the evidence was low. Therefore, delaying FET may unnecessarily delay pregnancy. Clinical decision-makers can consider patient preferences when selecting an appropriate time for FET after canceling fresh ET and menstruation. However, more future research is needed to confirm this finding.

## Data availability statement

The original contributions presented in the study are included in the article/Supplementary material, further inquiries can be directed to the corresponding authors.

## Author contributions

Y-QG: Writing – original draft. J-YS: Conceptualization, Formal analysis, Writing – review & editing. Z-GS: Funding acquisition, Supervision, Writing – review & editing.
