# Supplementary material for: The optimal timing of frozen-thawed embryo transfer: delayed or not delayed? A systematic review and meta-analysis
Source: Front Med (Lausanne). 2024 Jan 15;10:1335139. doi: 10.3389/fmed.2023.1335139 (PMC10825964; doi:10.3389/fmed.2023.1335139)
Supplement: Supplementary file 1 [file Data_Sheet_1.docx]

**Search strategy**

(freeze all) OR (fresh embryo transfer)) OR (infertility)) AND (frozen embryo transfer)) OR (frozen-thawed embryo transfer)) OR (cryopreserved embryo transfer)) AND (immediate)) OR (delayed)) OR (postpone)) AND (timing)) OR (time)) OR (time interval)) AND (oocyte retrieval)) OR (ovum pick-up)) OR (ovarian stimulation)) AND (IVF)) OR (Fertilization in Vitro)) OR (OPU)
